# Cell Salvage in the Management of Postpartum Hemorrhage: A Comprehensive Review of the Current Literature

**DOI:** 10.7759/cureus.95782

**Published:** 2025-10-30

**Authors:** Sara Giuliano, Daniela Ruiz, Nidhi Basavaraj, Guljabin Sultana, Rawnak Jahan, Thandar Aung, Olushola Ariyo, Esra Ahmed, Deeba S Zubair, Ramsha Ali

**Affiliations:** 1 Obstetrics and Gynecology, Università Cattolica del Sacro Cuore, Rome, ITA; 2 School of Medicine, Universidad Autónoma de Guadalajara, Guadalajara, MEX; 3 School of Medicine, JSS Medical College, Mysore, IND; 4 Obstetrics and Gynecology, GS Medical College, Pilkhuwa, IND; 5 Obstetrics and Gynecology, Bangladesh Medical College, Dhaka, BGD; 6 School of Medicine, Jinzhou Medical University, Jinzhou, CHN; 7 Obstetrics and Gynecology, North Middlesex University Hospital, London, GBR; 8 School of Medicine, University of Khartoum, Khartoum, SDN; 9 Obstetrics and Gynecology, Alshifa Multispeciality Hospital, New Delhi, IND; 10 School of Medicine, Peoples University of Medical and Health Sciences For Women, Nawabshah, PAK

**Keywords:** autologous blood transfusion, cell salvage, cesarean section, maternal outcomes, postpartum hemorrhage, vaginal delivery

## Abstract

Postpartum hemorrhage (PPH) remains a leading contributor in maternal illness and death worldwide, and its incidence continues to rise. This has led to an increased reliance on allogeneic blood transfusions (ABT), which are limited in supply and carry significant health risks. Cell salvage (CS), which collects and transfuses a patient’s own blood, has been proposed as a useful adjunct or alternative. This narrative review aims to evaluate and summarize the current literature on obstetric CS (OCS) in the management of PPH, with a focus on efficacy, safety, cost-effectiveness, and future research considerations. A literature search was performed using PubMed and Google Scholar, leading to the selection and review of 32 original articles published from 2015 onward. Single case reports, meta-analyses, and other review articles were excluded to minimize amplification bias and ensure an authentic analysis of primary research data. OCS has been shown to reduce the need for donor blood, especially during high-risk cesarean sections, including those with placenta previa and placenta accreta spectrum. Reported benefits include improved maternal hemoglobin, reduced transfusion-related complications, and shorter hospital stay. However, not all the results were statistically significant for reductions in allogeneic transfusion rates. Safety concerns associated with the use of OCS, including amniotic fluid embolism, sepsis, hemolysis, coagulopathy, and Rh alloimmunization, have been substantially addressed through advancements in technology, the implementation of leukocyte depletion filters, and the routine administration of anti-D prophylaxis. Evidence for its use in vaginal deliveries remains sparse, though early studies suggest technical feasibility. Cost-effectiveness is debated and appears dependent on institutional protocols, case volume, and technique optimization. OCS represents a safe and valuable blood-conservation strategy in obstetric practice, particularly for high-risk cesarean section. Wider adoption requires standardized protocols, staff training, and further multicenter research to clarify its role, especially in vaginal deliveries.

## Introduction and background

Introduction

Obstetric hemorrhage is a leading cause of maternal morbidity and mortality. Over the last decade, the incidence of postpartum hemorrhage (PPH) has risen in developed countries [1], resulting in higher demand for allogeneic blood transfusion (ABT) as part of its management. International expert committees and health organizations have established guidelines for the containment and treatment of PPH.

The World Health Organization (WHO) recommends monitoring uterine tone throughout labor and administering uterotonics, with oxytocin regarded as the gold standard, during the third stage of labor as primary strategies for the prevention of PPH. This is often paired with tranexamic acid (TXA) and controlled cord traction (CCT) to aid placental delivery. Additional approaches to manage or assess PPH include administering prostaglandins, mechanical tamponade, or performing artery embolization, reserving hysterectomy only when other measures fail [2]. Prompt restoration of blood volume is vital in the management of PPH. This is achieved through the administration of intravenous (IV) fluids or by implementing standardized transfusion protocols using allogeneic blood products as primary therapeutic interventions. Persistent disparities between blood donation rates and clinical demand continue to exert significant pressure on the availability of allogeneic blood supplies [3,4]. Complications of ABT can increase the length of hospital stay rates by 1.3% per unit transfused [4]. Other risks associated are, but not limited to, transfusion-related acute lung injury (TRALI), immunosuppression, transmission of viral infections [5,6], and various transfusion reactions, directly contributing to the rising costs associated with allogeneic blood use [7].

Cell salvage (CS), but more specifically obstetric CS (OCS), has emerged as a valuable alternative or adjunct to allogeneic blood transfusion in managing severe blood loss during obstetric procedures. While it does not eliminate the need for ABT, it has been shown to significantly reduce dependence on donor blood, thereby helping conserve the resource [8,9]. This method entails collecting blood from the operative area, followed by centrifugation, washing, filtration, and reinfusion into the patient. This process enables autologous transfusion. 

Despite endorsement in clinical guidelines in both the United Kingdom and the United States [10], CS implementation remains a subject of ongoing debate. Over the past three decades, significant advances in the technology and safety of CS have been made. However, potential complications remain a concern.

OCS has demonstrated both safety and effectiveness in controlled surgical settings; however, its application, especially during vaginal delivery, remains limited due to persistent concerns regarding complications such as amniotic fluid embolism (AFE), Rh alloimmunization, sepsis, and coagulopathies [11,12]. Despite its potential to improve maternal outcomes, research into the use of OCS for reinfusing blood lost during vaginal birth has stagnated, with few primary studies addressing its feasibility and safety in this context. Broader adoption is further hindered by practical challenges, including logistical barriers, cost considerations, the absence of standardized protocols, and ongoing safety debates. While isolated studies suggest promising clinical benefits, the evidence remains fragmented and often lacks integration into wider obstetric practice [6,7], highlighting the need for a comprehensive synthesis of existing research to clarify the role of OCS within contemporary PPH management.

Method

This narrative review aims to explore the role of cell salvage in the management of PPH and critically assess the currently disintegrated data to provide an overview of the present state of obstetric cell salvage. Specifically, it examines the current literature on efficacy, safety, cost-effectiveness, and practical considerations, with the goal of clarifying the value of OCS as a supportive strategy in obstetric bleeding management. A search of the PubMed and Google Scholar databases yielded 539 articles. Following the removal of duplicate entries, 332 unique articles remained for further analysis. Articles published prior to 2015 were excluded to ensure that our research reflects the most current information while adhering to established standards in the management of PPH. Single case reports were omitted due to their restricted analytical strength, which limits their capacity to contribute meaningful evaluations of patterns or trends. To ensure exclusive analysis of primary data, meta-analyses and reviews on OCS were excluded. Data was extracted from 75 articles, from which a grand total of 32 primary studies were selected and included in our review. Additionally, 10 guideline sources and one review on patient blood management were included. 

## Review

Cell salvage: an overview of the technique

CS is a multiphase process that allows for the collection, processing, and reinfusion of a patient's own blood, serving as an autologous alternative to allogeneic transfusion. It is commonly indicated in surgical patients expected to lose more than 500 mL of blood, or approximately 10% of their estimated blood volume, and is particularly beneficial for individuals with rare blood types, multiple alloantibodies, or those who decline donor blood for religious or ethical reasons [8,10]. Additionally, CS may be considered in patients with pre-existing anemia or in procedures associated with a high risk of intraoperative bleeding.

During the collection phase, blood is aspirated directly from the surgical site into a reservoir container or collected via rinsing of blood-soaked surgical swabs in heparinized normal saline. To prevent cell lysis, the aspiration is regulated at a low pressure (100-150 mmHg) and the surgical swabs are pressed delicately to rinse [10]. Owing to the heightened risk of a systemic inflammatory response, cardiovascular failure, and additional red cell lysis, agents such as non-IV antibiotics, iodine, chlorhexidine, topical clotting agents, fibrin-based glues, and orthopedic cements are both avoided and contraindicated during the collection phase [7,10]. To maintain optimal sterile conditions within the CS system, blood contaminated with urine or fecal matter is not aspirated. Sepsis and malignancy are not explicit contraindications for CS, but aspiration of purulent secretion is cautioned, and the collection during malignancy surgery has raised concerns over the need to irradiate the product before transfusing it back to the patient. The aspiration line is connected to the anti-coagulation agent, which is continuously delivered to the tip of the suction, preventing the blood from clotting before reaching the reservoir container. A filter in the collection reservoir removes large debris. 

**Figure 1:**
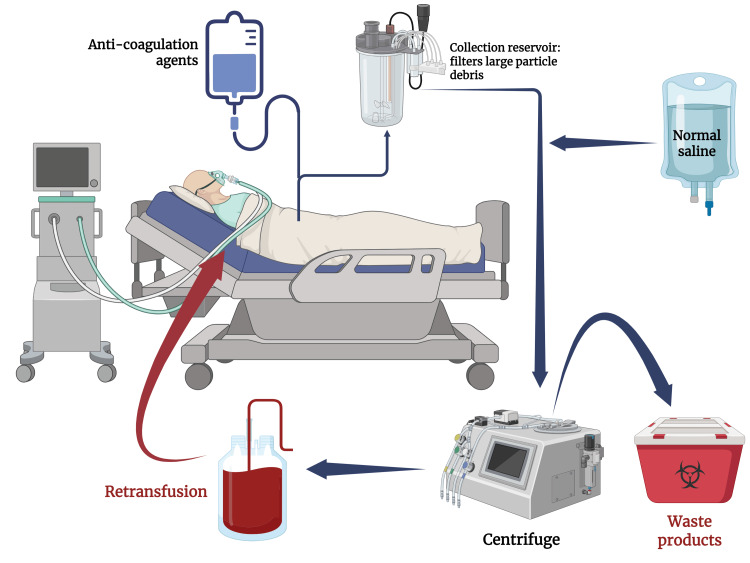
Cell salvage technique Created in BioRender. Abdin, Z. (2025) https://BioRender.com/4bvikpt

In the processing phase, the blood from the reservoir is washed with normal saline specific for IV administration. Once washed, centrifugation causes the red blood cells to separate from the rest of the blood components (plasma, anticoagulants, platelets, etc.). The isolated red cells are then washed again and prepared for the final phase of CS, transfusion back into the patient (Figure 1). The cell salvage method can also be used post-surgically for a short period of time if the patient loses sufficient blood through a wound drain. This blood can also be collected, processed, and then transfused back to the patient.

**Figure 2:**
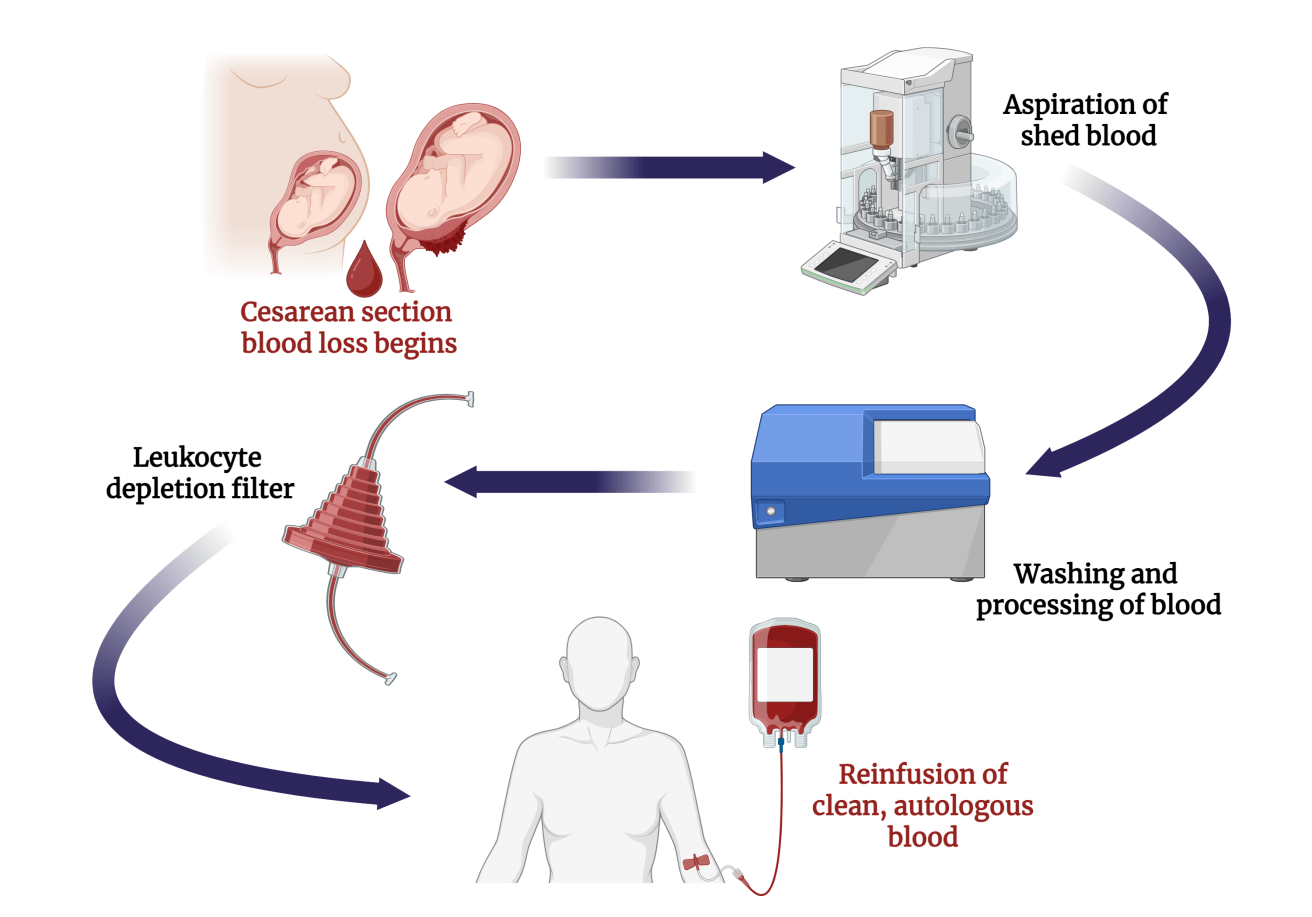
Cell salvage in cesarean section Created in BioRender. Abdin, Z. (2025) https://BioRender.com/i5e8xe0

In the context of OCS, the overall phases of the technique remain unchanged, but specific modifications are made to accommodate the presence of heavy bleeding, amniotic fluid, or other potential contaminants. The collection phase initiates after the delivery of both the baby and the placenta via caesarean section, and a second aspirator can be implemented for suction of amniotic fluid or impurities, while the primary aspirator is kept exclusively for suction of blood [2]. The dual-aspirator system can also be introduced in the event of acute massive hemorrhage, as a mode of collecting blood promptly and efficiently [13]. Once processed, salvaged blood can be reinfused with the use of leukocyte depletion filters (LDFs) to aid in further decreasing the number of contaminants (Figure 2) [7].

While CS is commonly associated with surgical procedures, its application extends to non-surgical settings. In obstetrics, a vaginal delivery may present a clinical scenario where this blood conservation method might be warranted. The cell salvage equipment is primarily located in the surgical theatre, making prompt access challenging during vaginal delivery. Therefore, optimal collection of vaginally-shed blood should ideally commence in the delivery room, utilizing sterile under-buttock pouched drapes to facilitate subsequent aspiration of heparinized blood [6]. Rinsing of swabs used during the procedure amplifies the red cell volume in the reservoir. Processing and reinfusion proceed as in a caesarean section, but due to increased concerns over sepsis, prophylactic antibiotics could be indicated following the reinfusion of vaginally-shed blood.

Safety considerations in OCS

**Figure 3:**
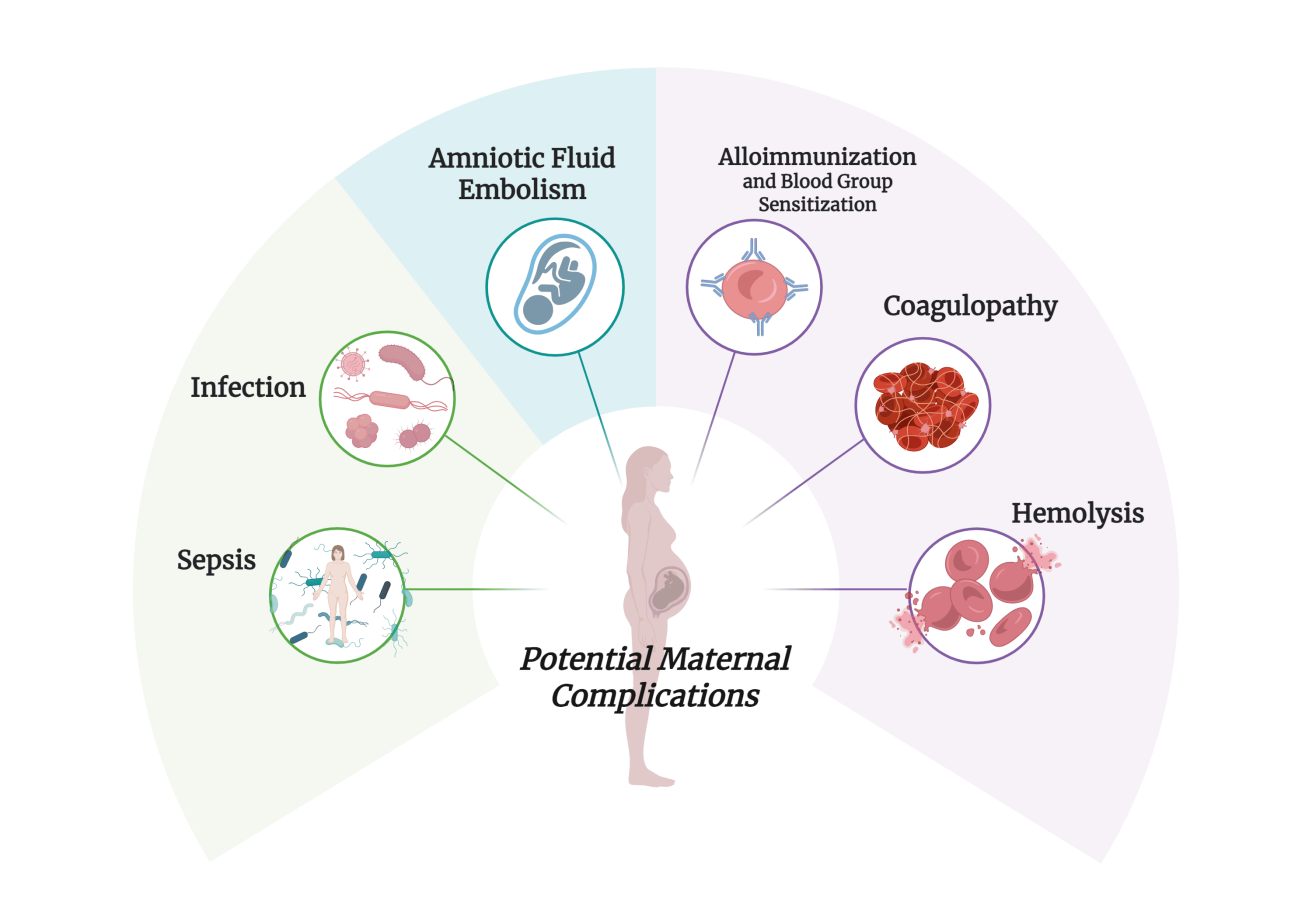
Maternal complications in obstetric cell salvage Created in BioRender. Abdin, Z. (2025) https://BioRender.com/7md81ss

As with any medical procedure, risks and safety concerns must be thoroughly addressed and reconciled to enhance its safety profile and subsequent applications. Historically, the adoption of OCS has been hindered by increased concerns of AFE, maternal alloimmunization, coagulopathy, and sepsis (Figure 3). In OCS, maternal safety is of paramount interest. In general, CS has demonstrated its safety and efficacy across multiple disciplines, as was observed in a large retrospective analysis of 33,351 patients who received salvaged blood, with only two presenting reversible adverse reactions associated with the procedure [14]. 

A review of data indicates that AFE has been the primary risk associated with OCS since its implementation. For years, the mechanism for AFE was not well understood. For instance, despite evidence supporting OCS safety in cesarean section, Morikawa et al. observed that while no adverse effects were presented with OCS, its safety remained unconfirmed due to the inability to truly assess AFE incidence [15]. It is now safely recognized that amniotic fluid contaminates salvaged blood with fetal squamous cells and other fetal elements, and although this does not cause a true embolic event, it can trigger a systemic inflammatory response leading to vasoconstriction, maternal hemodynamic instability, and cardiovascular collapse [16]. The inflammatory response can successively activate the coagulation and fibrinolytic pathways, resulting in enhanced PPH and disseminated intravascular coagulation (DIC). A cohort study assessing the adverse effects of OCS noted one case of AFE, where a patient developed cardiogenic shock and organ failure after receiving salvaged blood. However, the diagnosis was rebutted when it was affirmed that prior to CS, the patient had received allogenic blood products [12]. Despite concerns, recent studies have not demonstrated an incidence of true AFE during implementation of OCS [11,17].

A recent clinical study conducted by Rong et al. evaluated single versus dual-aspiration techniques to assess the impact of amniotic fluid and fetal cell debris following washing and filtration of salvaged blood [17]. The findings indicate that there is no statistically significant difference between the two techniques in the quantity of residual amniotic fluid markers, thus proving the effectiveness of CS washing and the use of leukocyte depletion filters in reducing amniotic fluid contaminants. 

During OCS, variable amounts of fetal red cells may be reinfused into the mother. This occurs due to the comparable size and density of fetal and maternal red cells, which prevents the CS equipment from differentiating them [18]. Consequently, despite thorough processing, fetal red cells can still be present in the blood, posing a concern only when there are incompatibilities of red cell antigens. To prevent maternal Rh alloimmunization, anti-D immunoglobulin prophylaxis should be administered according to established protocols. Appropriate use of anti-D prophylaxis during or after pregnancy effectively mitigates this risk. However, as noted by Sullivan et al., subsequent pregnancies may still be impacted, not only by anti-D antibodies but by the development of additional antibodies to other incompatible red cell antigens [18]. The development of anti-K, anti-c, anti-Jk, and anti-Fy antibodies remains a potential concern for fetal hyperbilirubinemia and anemia in later pregnancies, yet further research is required to understand their immunogenic strength. One tertiary obstetric unit recorded maternal antibody status and detected one case out of 436 of OCS alloimmunization with anti-D antibody in a subsequent pregnancy that was treated accordingly [19].

Concerns regarding coagulopathy as a complication of OCS remain, chiefly attributable to factors such as hemodilution related to substantial autotransfusion in massive PPH and hemolysis resulting in DIC. Wang et al. [9] and Zeng et al. [13] discuss hemodilution coagulopathy, noting that it arises not as a direct consequence of OCS but rather from excessive volume replacement leading to a reduction in clotting factors. Salvaged blood primarily contains red cells, with minimal clotting factors and platelets. This condition may occur with both OCS and ABT. In their respective studies, comparing coagulation factors between OCS and ABT, both found insignificant differences between the two groups pre- and post-operatively and did not observe any adverse reactions. A recent retrospective analysis of coagulation function indicated that both OCS and ABT resulted in prolonged aPTT and PT, though these values remained within normal ranges. However, OCS was associated with less pronounced changes and fewer adverse reactions [20]. OCS adheres to operational standards designed to ensure proper collection to maintain red cell integrity, thereby preventing hemolysis. Modern cell salvage equipment is engineered to minimize red cell damage, resulting in rare occurrences of clinically significant hemolysis. Clinical practice requires monitoring coagulation profiles and supplementing with plasma and platelets as indicated.

The potential risk of sepsis associated with reinfusion of salvaged blood is a notable concern, especially when blood shed vaginally is reintroduced [6,11,12,21]. This is primarily due to the challenges in maintaining a sterile environment and the proximity of the collected blood to the skin and genital region. Teare et al. evaluated the bacterial load present in vaginally shed blood following washing and determined that its profile was comparable to that of surgically collected blood, with predominant organisms *Escherichia coli*, *Enterococcus* species, and coagulase-negative *Staphylococci* [6]. A current descriptive analysis on a cohort of 64 patients, who received salvaged blood from a vaginal delivery, showed an absence of sepsis and attributed the results to improved washing and filtration technology, which markedly reduced bacterial load [11]. 

The literature supports the safety of OCS and consistently addresses concerns about its associated risks with evidence-based findings and recent data. In support of these safety considerations, a large multi-center observational study by Lyu et al. considered 2,621 patients across 12 hospitals for serious adverse effects following cell salvage, and no such events were found [22]. Furthermore, they developed a bleeding risk scoring system based on independent risk factors in obstetric patients to identify individuals at elevated risk of intraoperative bleeding (Table 1). This approach facilitates the selection of suitable candidates for cell salvage, thereby promoting a more efficient and comprehensive clinical application of OCS.

**Table 1:** Assessment of bleeding risk (blood loss volume ≥ 1500 mL) in OCS patients Adapted from: Lyu et al. [22]; licensed under a CC BY 4.0 License

Risk Factors	Points
Maternal age ≥ 35	+1
Prior cesarean sections	+1
Placental attachment position (abnormal)	+1
Placenta previa	+1
Placenta accreta (or spectrum)	+2
Blood pool in the placenta (ultrasound sign)	+2
Abnormal retroplacental myometrium (ultrasound sign)	+2
Placenta protruding to anterior wall	+2
Continuous disruption of myometrium	+2
Cervical canal invasion	+4
Maximum total points (maximum risk)	18 points
Optimal threshold / cut-off value	5 points

Indications and clinical outcomes of OCS

CS has repeatedly proven to be safe and effective in a range of elective adult surgical procedures, such as cardiac, orthopedic, vascular, and transplant operations, where its use is frequently standard practice. With respect to its obstetric indications, OCS is increasingly acknowledged, albeit gradually, as a valuable adjunct to conventional blood transfusion in the management of PPH, particularly during cesarean section, both elective and emergent. It is further recommended for deliveries already expected to result in substantial blood loss, such as those involving placenta previa, placenta accreta, or ruptured ectopic pregnancy [3]. 

Tanaka et al. highlight a specific clinical indication of OCS in the management of patients who decline ABT, such as those belonging to the Jehovah’s Witnesses faith or individuals with similar personal or ethical convictions [23]. Refusing allogeneic blood transfusion, regardless of the obstetric risks involved, constitutes a significant risk in its own right. It should be emphasized that the mortality rate among Jehovah's Witnesses due to obstetric complications is 6-65 times higher than that observed in the general obstetric population [23].

Most of the studies reviewed focused on the use of CS within controlled surgical obstetric environments; however, only one study in the past 10 years examined its application in vaginal deliveries. In 2015, four studies were conducted to investigate OCS (Table 2) [6,7,15,24]. 

**Table 2:** Studies on indication, application, and outcomes for obstetric cell salvage ABT: allogenic blood transfusion; CS: cell salvage; C/S: cesarean section; ICS and IOCS: intraoperative cell salvage; OCS: operative cell salvage; JW: Jehovah's Witnesses; PAS: placenta accreta spectrum; RTC: randomized control trial

Title	Year of publication	Study design	Study objective	Population	Conclusion	Limitations	Authors
Intraoperative cell salvage as an effective intervention for postpartum hemorrhage: evidence from a prospective RCT	2020	Prospective RCT	Evaluate the efficacy and safety of ICS as a monotherapy in patients with a high risk of PPH.	65 interventions 65 controls	ICS maintained coagulation function and increased Hb levels with greater efficacy than ABT. ICS did not present with adverse reactions.	It is a single-center study. Only 130 patients in total.	Lei et al. [4]
Is cell salvaged vaginal blood loss suitable for re-infusion?	2015	Observational study	To evaluate OCS for vaginal deliveries	50 cases	The aspiration of blood to the cell salvage machine can be delayed. Re-infused blood would result in a circulating bacteremia of <1 CFU/mL	Small sample size	Teare et al. [6]
Implementation of an obstetric cell salvage service in a tertiary women's hospital	2015	Observational study	ICS for the management of massive obstetric hemorrhage in patients with PAS and placenta previa	11 cases.	No adverse events reported in ICS patients.	Very small sample size	Lew et al. [7]
Cell salvage and donor blood transfusion during cesarean section: A pragmatic, multicentre randomised controlled trial (SALVO).	2017	RCT	Comparison of routine CS and current standard of care during C/S.	1498 interventions 1492 to controls	Significant reduction of ABT in emergency C/S	The study does not provide long-term follow-up data on alloimmunization.	Khan et al. [8]
Intraoperative cell salvage is associated with reduced allogeneic blood requirements and has no significant impairment on coagulation function in patients undergoing cesarean delivery: a retrospective study	2020	Retrospective Cohort Study	Examine the association between ICS, ABT and coagulation function in obstetrics	101 interventions 56 controls	The use of ICS is associated with lower requirement rate for ABT (P < .001). No significant difference in coagulation function was observed between groups in preoperative and postoperative phase (P > .05)	Possible selection bias due to study design. Small sample size.	Wang et al. [9]
How about "The effect of intraoperative cell salvage on allogeneic blood transfusion for patients with placenta accreta": An observational study.	2018	Retrospective Observational Study	Effect of IOCS on rates of ABT during C/S for placenta accreta	108 interventions 115 controls	IOCS was associated with a lower incidence of ABT (OR, 0.179; 95% CI, 0.098–0.328). Coagulation function and the need for coagulation components showed no significant difference between groups	This is a retrospective design. Selection bias may exist for the subjects who were not randomly allocated.	Zeng et al. [13]
Intraoperative red cell salvage during obstetric surgery in 50 Japanese women	2015	Retrospective cohort study	Evaluate the safety and effectiveness of ICS in obstetric surgery among Japanese women.	50 cases	In 50 cases, cell salvage was feasible, reducing the need for allogeneic transfusion without major complications.	The present study does not prove that ICS during cesarean delivery is safe.	Morikawa et al. [15]
Obstetric outcomes and acceptance of alternative therapies to blood transfusion by Jehovah's Witnesses in Japan: a single-center study.	2018	Retrospective study	The acceptance rate of alternatives to blood transfusion. To examine obstetric outcomes of delivery in JW patients.	84 cases	52.4% accepted all blood products and all types of autotransfusion. ICS was accepted by n57 67.9%	Only 5 people in the whole study got ICS during the study, and these 5 pt were cases with severe obstetric complication.	Tanaka et al. [23]
Red blood cell salvage during obstetric hemorrhage.	2015	Retrospective cohort study	Looking at the data of PPH cases where CS was used	884 cases	Only 13% of routine c-section patients received an intraoperative blood salvage reinfusion. Only 53% of the patients who bled after vaginal delivery received an intraoperative blood salvage reinfusion (P < .001)	Only mentioned PPH from vaginal deliveries.	Milne et al. [24]
ICS as part of a blood conservation strategy in an obstetric population with abnormal placentation at a large Irish tertiary referral center: an observational study.	2020	Retrospective cohort study	IOCS use and ABT patterns in patients with abnormal placentation.	139 cases	The re-transfusion rate was 18.5%. Five patients received IOCS blood only. The allogeneic transfusion rate was 7.5% in patients who had IOCS setup compared with 6.9% in those who did not (p = 1.00).	Results not statically significant.	O'Flaherty et al. [25]
Use of intraoperative red cell salvage in the contemporary management of placenta accreta spectrum disorders.	2023	Retrospective observational study	investigated hemorrhagic and non-hemorrhagic morbidity in a cohort of patients treated after the routine implementation of IOCS during Cesarean hysterectomy	119 cases	The use of IOCS was associated with low overall incidence and volumes of allogenic blood product transfusion, intraoperative hemorrhagic morbidity, and subsequent consumptive coagulopathy, achieving complication rates that are lower	Safety for AFE was not investigated. Patients were not followed prospectively. Possible unmeasured confounding variables not considered	Flores-Mendoz et al. [26]
Intraoperative cell salvage for obstetrics: a prospective randomized controlled clinical trial.	2020	RCT	To evaluate the clinical value of intraoperative autologous blood cell transfusion in obstetric surgery.	80 interventions 80 controls	ICS significantly reduced: the amount of ABT, the incidence of adverse effects, and hospital stay in patients with severe bleeding.	Exclusion criteria Hgb < 9g/dL. Maternal RhD-negative blood type	Liu et al. [27]
Association between routine cell salvage use for lower segment caesarean section and post-operative iron infusion and anemia.	2023	Cohort study	Examine the association of routine ICS and rates of postpartum anemia and IV iron.	431cases	Post-operative Hgb was higher and anemia cases fewer in the ICS group. Rates of post-partum iron infusion were significantly lower in the ICS group (OR =0.31, 95% CI=0.12 to 0.80, P=0.016)	Only 1pt for each groups got pRBCs	Fox et al. [28]
Impact of cell salvage on hematocrit and post-partum anemia in low hemorrhage risk elective cesarean delivery.	2024	Cohort study	To assess the impact of ICS on hemoglobin and postpartum anemia after low hemorrhage risk cesarean delivery.	99 cases	CS increased postoperative hemoglobin and reduced postpartum anemia without added complications.	Small sample size	Katz et al. [29]

In 2017, Khan et al. conducted a randomized controlled trial involving over 3,000 women undergoing cesarean delivery at risk of hemorrhage to assess the efficacy of OCS compared with standard care [8]. Their trial demonstrated a lower proportion of women in the OCS group requiring allogeneic blood transfusion (2.5% vs 3.5%); however, this difference did not achieve statistical significance. No cases of amniotic fluid embolism were reported, irrespective of the use of leukocyte depletion filters. A notable limitation of the study was the absence of long-term assessment of RhD-negative women with respect to their postpartum RhD status, particularly given the concurrent finding of increased maternal exposure to fetal blood.

Zeng et al. investigated the use of intraoperative obstetric cell salvage (IOCS) in cesarean deliveries among women with placenta accreta spectrum, reporting favorable outcomes [13]. In cases with an estimated blood loss ≤ 3000 mL, ABT was avoided in 93.0% of patients in the IOCS group, whereas 50.0% of the control group required ABT. Among women with blood loss > 3000 mL, reinfusion of IOCS blood appeared to reduce transfusion requirements in six of 21 patients (28.6%), compared with universal need for ABT among the 17 controls. Importantly, no significant differences were observed between the two groups in terms of coagulation function or the requirement for coagulation components (P > .05). 

Lei et al. have proven that OCS transfusion effectively improves the hemoglobin level in those patients who were classified at a high risk of PPH and are hence indicated to undergo elective or emergency cesarean section [4]. Clinical scenarios associated with a high risk of PPH include placenta previa, placenta accreta, and complicated cesarean sections. Among these high-risk scenarios, in 2015, Lew et al. reported 11 cases, of which ICS with reinfusion was applied only to four; no adverse events were observed in these patients [7]. However, the small sample size (n = 11) limited the ability of the authors to assess the effectiveness of ICS in reducing the need for ABT. In 2020, O’Flaherty et al. aimed to investigate IOCS use in patients with a diagnosis of suspected PAS (patient cohort from 2015), who were, by definition, deemed at high risk of hemorrhage [25]. Their conclusion stated that IOCS contributed to a reduction of allogeneic transfusion for high-risk patients; however, it must be pointed out that their results did not reach statistical significance. Similarly, Flores-Mendoza et al. conducted a retrospective observational study investigating the implementation of IOCS for cesarean section in patients with high suspicion of PAS [26]. The use of IOCS was associated with low overall incidence and volumes of allogeneic blood product transfusion, intraoperative hemorrhagic morbidity, and subsequent consumptive coagulopathy, achieving complication rates that are lower than those reported in other studies. The study by Wang et al., consistent with several other studies, assessed the impact of ICS on the requirement for ABT, resulting in the use of ICS being associated with a lower requirement rate for ABT (P < .001) [9].

Liu et al. emphasize further benefits of ICS in high-risk PPH population, reporting a reduced amount of ABT, lower incidence of postoperative wound infection, delayed wound healing, perioperative allergic reaction, adverse cardiovascular events, hypoproteinemia, and a shorter duration of hospital stay among patients with severe bleeding [27]. A major limitation of this study was the exclusion of RhD-negative women, which prevented evaluation of one of the principal concerns regarding the implementation of OCS.

Only one study by Teare et al. assessed the suitability of implementing OCS during vaginal delivery, demonstrating that blood can be collected efficiently with minimal impact or disruption to patient management, but more importantly, after the midwife has poured the heparinized saline, after recognition of the excessive bleeding, the aspiration of blood to the cell salvage machine can be delayed until trained personnel arrives [6]. Regarding the risk of infection, bacteria are yet detectable, but if re-infused, would result in a circulating bacteremia of <1 CFU/mL, which is similar to what is seen with a dental procedure. Additionally, results from this study are consistent with those obtained from a study conducted in 2008 by Sullivan et al. [30].

Fox et al. in 2023 [28] and Katz et al. in 2024 [29] investigated the impact of OCS on post-partum hemoglobin and hematocrit values, as well as postoperative iron infusion. Both authors concluded that CS indeed increased postpartum hemoglobin level and reduced the rate of postpartum anemia. It must be pointed out that these studies did not investigate the impact of CS on the need for ABT.

Outlook on cost-effectiveness and protocol improvement to mitigate limitations

OCS offers insurmountable benefits over allogeneic blood transfusion, yet it continues to be examined on the matter of immediate, perceptible cost rather than its fundamental indication as a medical resource for improved patient outcome and global relief. OCS is an advanced novel resource whose outlook is unjustifiably influenced by institutional economics, as was demonstrated by one of the largest, yet insufficiently diverse, randomized controlled trials (RCTs), which concluded that routine OCS did not prove to be cost-effective [8], possibly limiting global use of OCS. Lim et al., who also analyzed cost, reached the same conclusion but suggested a more collective approach to the outlook on cell salvage for obstetric use, calling for a differentiation between “cost-saving” and “cost-effective” strategies that would allow for the fair individualization and proper allocation of CS despite the possibility that the procedure might “not save money” [31].

Through data analysis, it can be reasoned that the cost-utility and perceived effectiveness of OCS rely on case volume and the amount of blood retrieved for successful processing [7,32]. Given the fact that obstetric and postpartum hemorrhage are highly unpredictable, even in apparently healthy women, the increase in case volume can be achieved if new applications are explored by considering cases with lower expected blood loss, vaginal deliveries, and emergent cases occurring after-hours [11,25]. Leeson et al., through their cohort study, suggest that the routine use of OCS may be advantageous, since it has been reported that 60% of patients with PPH had no identifiable risk factors prior to onset [19]. 

By optimizing blood collection techniques using the “collect only” or “standby” mode, OCS can be indicated without the increased cost implication if insufficient blood is collected for processing. In this modality, the setup includes only the aspirator, reservoir, and anticoagulation line, without integrating the costly processing phase of cell salvage. The associated costs of the standby mode approximate those of the reagents needed for the crossmatching of two allogeneic blood units [25]. Technical improvements like the adaptation of a dual aspiration system, a sponge rinsing network, or the effective use of sterile pouched drapes can significantly increase the red cell yield [33], thereby increasing the likelihood of obtaining sufficient blood to advance the procedure. Data on the gravity-driven HemoClear microfiltration CS system, although biased, have emerged as an economically feasible alternative to centrifugal CS, while fostering the possibility of platelet salvage in the future [34]. 

Certainly, there are methods that can decrease the financial risks of OCS through the effective administration of consumables and supplies, as well as staff. The effectiveness of OCS and its overall stance as a blood conservation method is conveyed in its clinical applications, not its cost. Vigorous refinement and heightened trust of OCS protocols need to occur as well. The decision to utilize CS may subjectively depend on a clinician’s expertise on the equipment itself, which oftentimes is stigmatized as unfamiliar and time-consuming equipment [35], reserved only for crisis situations. Protocols for OCS should undoubtedly give clinicians confidence in decision-making in the event of massive transfusion protocol (MTP) activation, but also in a comprehensive scope, in the face of ambiguity over the expected blood loss [25].

**Figure 4:**
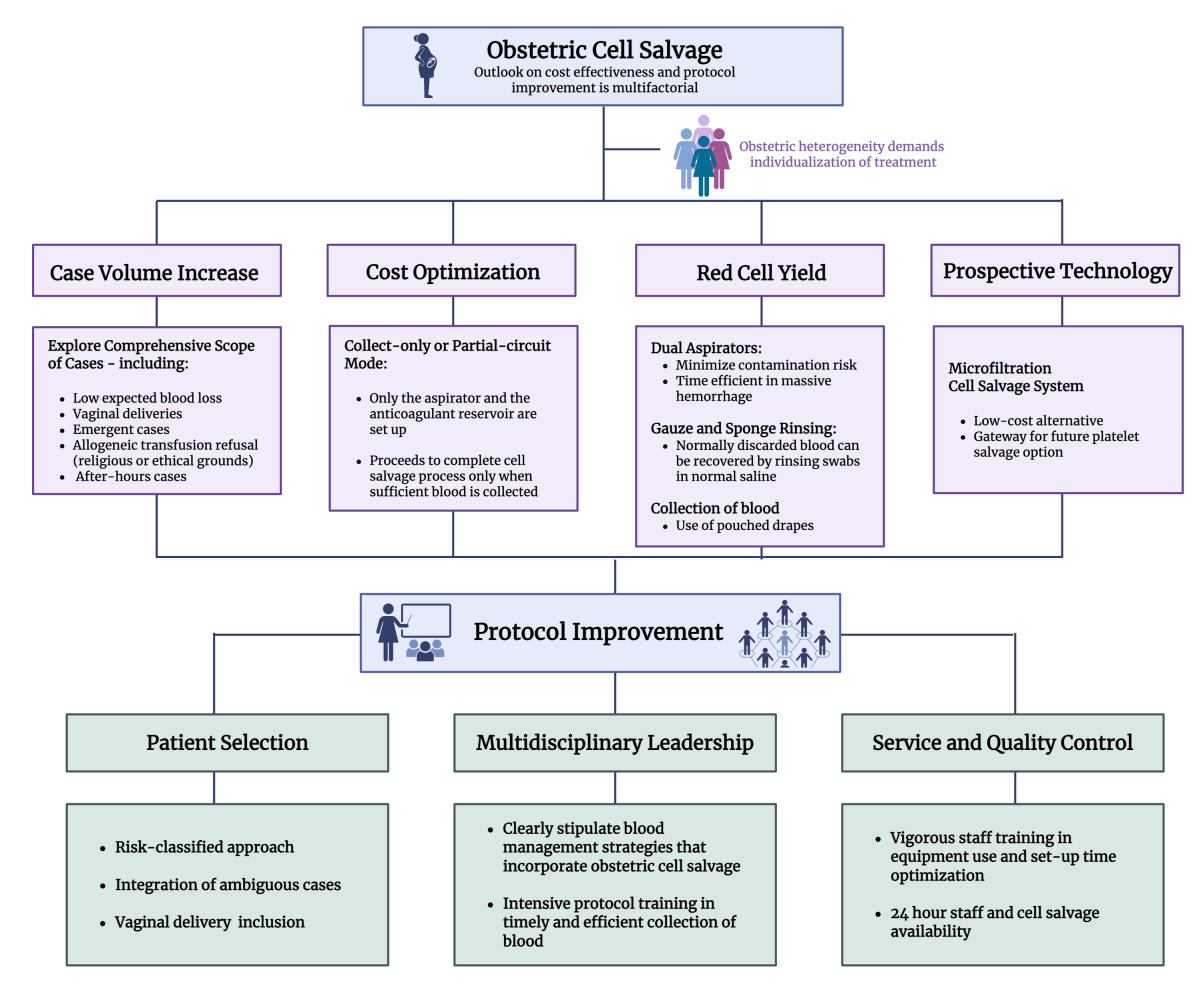
Multidisciplinary approach to cost-effectiveness and protocol improvement of obstetric cell salvage Created in BioRender. Abdin, Z. (2025) https://BioRender.com/dzhajsm

It is the unconventional cases, those most excluded from trials on cost-effectiveness, that offer insight into the heterogeneity of obstetrics where OCS can be safely applied [25], but cases are frequently overlooked or improperly managed. Phillips et al. conducted a retrospective analysis of OCS in vaginally shed blood, reporting a median blood loss of 2175 mL [11]. Almost 90% of cases presented blood loss greater than 1000 mL, with patients receiving, on average, 384 mL of salvaged blood product. It was noted that patients with severe vaginal hemorrhage were transferred to the operating room for further management. However, OCS blood collection commenced only after the patient arrived in the theater, resulting in the discarding of blood that was shed in the delivery room. This matter was attributed to inadequate protocol development, which resulted in high blood loss with low volume of autotransfusion. OCS protocols require input from multidisciplinary staff, strict competency training, and quality control. Uninterrupted OCS service is required to ensure full patient care [10] and true assessment of efficacy. The outlook on the cost effectiveness of OCS is multifactorial and relies, in part, on the adequate development of its protocols to expand its use, lower its perceived cost burden, and attenuate its limitations (Figure 4).

Guidelines and recommendations from professional bodies 

During the past decade, professional societies in Europe, Australia, and the United Kingdom have gradually harmonised their recommendations regarding the management of severe hemorrhage [10,36-41]. However, these guidelines have not yet been comprehensively or unequivocally aligned to incorporate cell salvage methods. 

Regarding PPH, the updated German, Austrian, and Swiss guideline highlights the need for every obstetric unit to establish a clear, multidisciplinary protocol that combines surgical, medical, and hemostatic interventions from the earliest stages of bleeding [36]. The emphasis is on anticipation and coordination: rapid administration of uterotonics, early use of tranexamic acid, structured coagulation monitoring, and, where needed, massive transfusion protocols.

Similar priorities are echoed in the 2024 Australian guideline on critical bleeding, which underscores the importance of major hemorrhage protocols as standard of care [42]. These protocols are not just about transfusion ratios, but about ensuring timely monitoring of physiological, biochemical, and coagulation parameters. This guideline recommends at least a 2:1:1 ratio of red cells to plasma and platelets, discourages the use of recombinant factor VIIa outside research or exceptional circumstances, and endorses tranexamic acid within the first three hours of bleeding onset. Cell salvage is mentioned, but here the evidence remains less robust, and the guideline calls for further study before it can be fully integrated into routine practice.

In the United Kingdom, the Association of Anesthetists and partner societies have taken a strong position in support of IOCS as a blood conservation technique [35]. Their 2018 guideline recommends that CS should always be available in hospitals where patients undertake major surgery, and used whenever blood loss is expected to exceed 500 mL. In obstetrics, the recommendation is more cautious: routine use during caesarean section is not currently supported, but the technique is considered appropriate in “collect only” mode for women with anemia, anticipated high risk of hemorrhage or unexpected intraoperative bleeding, or when a woman declines autologous transfusion [10]. The hesitancy reflects longstanding concerns about contamination of salvaged blood with amniotic fluid or fetal cells. Yet recent reviews and experience from high-risk cases, such as placenta accreta spectrum disorders, have not reported confirmed cases of amniotic fluid embolism [13,26]. 

A multidisciplinary consensus statement developed by the Network for the Advancement of Patient Blood Management, hemostasis and Thrombosis (NATA) in collaboration with the International Federation of Gynecology and Obstetrics (FIGO), the European Board and College of Obstetrics and Gynecology (EBCOG) and the European Society of Anesthesiology (ESA) stated that Intraoperative cell salvage should be available in high-level maternal care centers and used in cases of massive PPH (level of evidence 1C) [37].

Retrospective analyses also suggest that, in women requiring transfusion at caesarean section, CS could reduce exposure to donor blood in up to a quarter of cases [43]. However, evidence from large trials such as SALVO reminds us that routine use in low-risk caesareans does not confer measurable benefit [8].

Future direction and research priorities

Looking ahead, future research should thoroughly and meticulously address the safety and effectiveness of CS in obstetrics. Although the collection of observational data yields valuable insight, well-structured multicenter randomized controlled trials with sufficient sample sizes remain essential in defining and validating the role of OCS in both high- and low-risk caesarean procedures. Furthermore, such trials are critical for assessing quality of life and potential long-term outcomes, including maternal alloimmunization. Greater emphasis is also warranted on the application of OCS during vaginal deliveries, whether for immediate or delayed reinfusion, given the incidence of PPH in this context. 

An ample evaluation of OCS entails intercontinental research with a diverse and heterogeneous sample population, accompanied by a global registry of outcomes to enable pattern identification and accurate assessment of cost-effectiveness. A developed nation that implements OCS in conjunction with consistent, reliable access to allogeneic blood products may have outcomes that differ significantly from those seen in less developed countries where donor blood availability is substantially limited, highlighting the importance of thorough and varied research.

## Conclusions

OCS is a valuable source in the management of obstetric hemorrhage, particularly in high-risk cases. It reduces reliance on donor blood, avoids transfusion-related risks, and is valuable for women declining allogeneic transfusion. Safety concerns such as amniotic fluid embolism, infection, and alloimmunization have been reduced by modern washing and filtration techniques, with current evidence supporting its use when guided by established protocols. Routine use in low-risk settings is not yet recommended. Future priorities lie in conducting multicenter trials, refining protocols, exploring cost-effectiveness in varied contexts, and extending applications to vaginal deliveries. With ongoing research and protocol optimization, OCS has the potential to become an integral component of patient blood management strategies in obstetrics, ensuring safer, more sustainable, and patient-centered care worldwide.
